# Personals

**Published:** 1919-03

**Authors:** 


### Personals.

Dr. George W. Cox of Ozona, Texas, has formed a partnership with Dr. A. G. Blanton, who has recently moved from San Angelo to Ozona.

Posing as Doctor Robert H. Milwee Jr., of Dallas, a man who accepted the commission of first lieutenant of the Medical Corps in the army, proffered Dr. Robert H. Milwee last summer, has just been arrested by Federal authorities in New Orleans.

Dr. Robert H. Milwee, who has just returned from the service as lieutenant, junior grade, in the navy, says that he did not accept the army commission last summer as he wanted to get into the navy. While in Washington last week he found that there are two names listed in the Government records. He has no idea who the alleged imposter is.

---

Doctor M. C. Bell of Brownfield has moved with his family to Abilene and will practice medicine there. He was until recently in the Medical Corps of the United States.

---

Miss Lena Clermont, formerly owner and superintendent of a hospital in Vancouver, B. C., has located in Ranger and will establish a modern hospital.

Miss Clermont is a graduate nurse and has made a close study of the profession in Canada, Australia and the United States.

In 1916 she was appointed by the British Government to train nurses in Vancouver for overseas service.

---

Doctor B. Y. Lacey of Pittsburg was appointed by Governor Hobby on the State Board of Medical Examiners.

---

At the regular meeting of the board of managers of the Texas State Lunatic Asylum the board re-elected Dr. John L. Preston as superintendent and Dr. Wilhite was re-elected to the Pasteur Institute.

---

"What is the plural of man, Willie?" asked the teacher of a small pupil.

"Men," answered Willie.

"And the plural of child?"

"Twins," was the unexpected reply.

